# Micro and Nanotechnologies Enhanced Biomolecular Sensing

**DOI:** 10.3390/bios3030283

**Published:** 2013-07-05

**Authors:** Tza-Huei Wang

**Affiliations:** Departments of Mechanical Engineering, Biomedical Engineering and Oncology, John Hopkins University, Baltimore, MD 21218, USA; E-Mail: thwang@jhu.edu; Tel.:+1-410-516-7086

**Keywords:** microfluidics, nanoparticles, quantum dots, disease diagnostics, DNA/RNA detection

## Abstract

This editorial summarizes some of the recent advances of micro and nanotechnology-based tools and devices for biomolecular detection. These include the incorporation of nanomaterials into a sensor surface or directly interfacing with molecular probes to enhance target detection via more rapid and sensitive responses, and the use of self-assembled organic/inorganic nanocomposites that inhibit exceptional spectroscopic properties to enable facile homogenous assays with efficient binding kinetics. Discussions also include some insight into microfluidic principles behind the development of an integrated sample preparation and biosensor platform toward a miniaturized and fully functional system for point of care applications.

The significant advances in micro and nanotechnologies have led to an increasing interest in developing biosensors engineered at the small length scale from molecular probes, nanomaterials and sensing structures as the basis for the next-generation biosensor and biosensing system to achieve higher sensitivity, specificity, throughput, portability and cost effectiveness. On the one hand, the emerging field of microfluidics to create so called lab-on-a-chip (LOC) promises exciting solutions for integrating sample preparation with biosensors to realize point of care (POC) diagnostics. Biosensing systems based on microfluidics allow processes that were previously done at the large scale and in separate operations to be miniaturized and integrated onto a single platform, improving both speed and efficiency. On the other hand, the advances in synthesis chemistries have allowed scientists to create monodisperse nanoparticles with controlled shapes, sizes and compositions, thereby providing a way to precisely manipulate material properties. Such development aims beyond improving upon current techniques in terms of speed and sensitivity, but strives to enable scientists to conduct studies and clinicians to conduct tests that were unfeasible or impractical with previous technologies. This special issue focuses on the contributions related to the development or use of micro and nanomaterials, devices, and techniques for biomolecular sensing research and applications [1,2,3,4,5].

Rapid identification of infectious agents is critical for management of infectious diseases as it provides timely therapeutic intervention that leads to saving life. Real-time polymerase chain reaction (PCR) has been gradually replacing traditional culture assays for infectious disease diagnostics because nucleic acids-based detection provides higher sensitivity and shorter turnaround time. However, the widespread use of PCR assays in clinics has been hampered by the tedious sample preparation process. In this issue, Packard *et al.* [1] described a microfluidic chip that incorporates on-chip capture and concentration of bacterial cells, thermal lysis, and fluorescence resonance energy transfer-assisted *in situ* hybridization (FRET-ISH) for species identification. This device facilitated a genetic-based detection of pathogens without the use of PCR. The entire analysis can be accomplished with 30 min which offers a great promise for POC applications. 

In recent years, researchers have demonstrated that nanoparticles can provide elegant solutions to applications ranging from compound screening, drug delivery and disease diagnostics. Such nanoparticles can generally be classified into semiconductor, metallic, and polymeric varieties. Each has their own unique physical, optical, and chemical properties that make them suited towards specific applications. Roh [2] reported the use of semiconductor quantum dot-conjugated RNA oligonucleotides for inhibitor screening of Hepatitis C virus NS3 protein. The study provides a simple and yet reliable platform for investigating target-compound interactions. Further improvements of this platform will contribute to efficient and effective methods for high throughput screening (HTS) for antiviral drug discovery. 

Electrochemical-based biosensors have played an essential role in the transition towards POC diagnostics because such devices are capable of delivering diagnostic information in a simple and low cost fashion via compact analyzers. The coupling of electrochemical biosensors with nanoparticles can offer enhanced sensitivity and multiplexing capabilities. Lin *et al.* [3] demonstrated an iridium nanoparticle-mediated electrochemical immunosensors for detection of prostate cancer biomarkers in patient blood samples. This sensor was demonstrated to accurately distinguish between healthy control samples and patients with high grade prostatic intraepithelial neoplasia. 

DNA-templated metal nanoclusters have emerged as a new class of fluorescent molecular probes with several advantages over conventional organic dyes such as superior photostability and high brightness. Obliosca *et al.* [4] reviewed the recent development of DNA-templated silver nanoclusters for DNA/RNA detection. They discussed the basic design of silver nanoclusters as nucleic acid probes to enable on-and-off switch of fluorescence and tunable emission wavelengths with nearby DNA sequences. They also described a new molecular probe termed NanoCluster Beacons that fluoresce upon hybridization with targets, but do not use FRET as the signal transduction mechanism. Several other nanocluster-based DNA/RNA probe designs that take advantage of the environmental sensitivities of DNA/Ag nanoclusters for DNA/RNA detection as well as single nucleotide polymorphism (SNP) discrimination are also introduced. 

The use of biosensors can also be applied to the detection and monitoring of environmental pollutants including genotoxicants. Geng *et al.* [5] reported a fish cell biosensor system capable of genotoxicity detection in a high throughput manner. The sensor was developed by integrating two report plasmids into marine flatfish flounder gill cells and was validated by a p21-mediated luciferase reporter gene bioassay system for genotoxicity. It is believed that the fish cell biosensor may become a specific and sensitive tool for genotoxicity detection of new chemicals and drugs. 

While the development of micro and nanotechnological tools and devices for biosensing applications is still in its infancy, a variety of micro and nanoscale biosensors, as reviewed in this special issue, have demonstrated superior performance compared with conventional biosensors. Miniaturization, in addition to the extraordinary physical properties of materials at the nanoscale, has enabled new sensors to transduce signals large enough in amplitude and discrete enough in identity to be positively identified at the single molecule level. We expect that the next ten years will see a period of rapid maturation and growth in current applications and undoubtedly entail the discovery of new properties and applications that will define the future of micro and nanobiosensors in scientific studies and clinical practices.
